# Endurance Training Increases Leptin Expression in the Retroperitoneal Adipose Tissue of Rats Fed with a High-Sugar Diet

**DOI:** 10.1007/s11745-013-3854-7

**Published:** 2013-11-16

**Authors:** Karina Barbosa de Queiroz, Juliana Bohnen Guimarães, Cândido Celso Coimbra, Gisele Vieira Rodovalho, Cláudia Martins Carneiro, Elísio Alberto Evangelista, Renata Guerra-Sá

**Affiliations:** 1 Present Address: Laboratório de Bioquímica e Biologia Molecular, Departamento de Ciências Biológicas, NUPEB, ICEB2, Universidade Federal de Ouro Preto, Room 045 Morro do Cruzeiro Campus, Ouro Preto, MG 35400-000 Brazil; 2 Laboratório de Imunopatologia, Departamento de Ciências Biológicas, NUPEB, ICEB, Universidade Federal de Ouro Preto, Ouro Preto, MG Brazil; 3 Departamento de Fisiologia e Biofísica, Universidade Federal de Minas Gerais, Belo Horizonte, MG Brazil

**Keywords:** High-sugar diet, Chronic exercise, Leptin expression, Obesity, Lipids, Metabolism

## Abstract

The presence of leptin receptors in white adipose tissue (WAT) suggests a type of peripheral control during the development of obesity and other metabolic disorders. Both diet composition and exercise influence serum leptin; however, the effect of their combination on long-term WAT leptin metabolism is unknown. In this study, rats fed with standard or high-sugar diets (HSD) were simultaneously subjected to running training for 4- and 8-week periods, and the retroperitoneal WAT (rWAT) was evaluated for adipocyte cell size, lipid and catecholamine content, *Lep*, *OB*-*Rb* and *Ucp2* mRNA transcription levels, and circulating leptin and non-esterified fatty acids (NEFA). The HSD groups displayed a higher adiposity index and rWAT weight, *Lep* mRNA and protein upregulation, and a period-dependent effect on *OB*-*Rb* mRNA expression. Exercise decreased serum leptin and upregulated the *OB*-*Rb* mRNA levels. However, in rats fed with an HSD, the increase in *OB*-*Rb* mRNA and reduction in catecholamine levels resulted in a high level of adiposity and hyperleptinemia. The combination of training and an HSD decreases the NEFA levels and upregulating the *Ucp2* mRNA expression in the 4-week period, while downregulating the *Ucp2* mRNA expression in the 8-week period without changing the NEFA levels. Our results suggest that an HSD induces an increase in leptin expression in rWAT, while reducing adipocytes via leptin-mediated lipolysis after an 8-week period. In exercised rats fed an HSD, TAG synthesis and storage overlaps with lipolysis, promoting fat store development and *Lep* mRNA and plasma protein upregulation in adult rats.

## Introduction

Leptin, a cytokine mainly produced by white adipose tissue (WAT), is a 16 kDa protein that acts directly on hypothalamus, regulating energy homeostasis and adiposity by reducing food intake and increasing energy expenditure [1]. The hormone acts via transmembrane receptors (OB-R) expressed in the central nervous system and peripheral tissues [2]. There are six leptin-receptor isoforms known (OB-Ra, OB-Rb, OB-Rc, OB-Rd, OB-Re and OB-Rf), which are divided into three classes: long, short and soluble [3, 4]. The long spliced OB-Rb variant mediates the effects of leptin via the JAK-STAT signaling pathway [5, 6], and the soluble OB-Re isoform acts as a leptin-linked circulating protein that modulates the steady state of the hormone by preventing its degradation and clearance [3, 7].

The leptin actions in the nuclei hypothalamic has been exclusively related to its ability to regulate food intake, body weight, adiposity and insulin sensitivity [1, 8]. However, the presence of leptin receptors in others tissues (for instance, adipose tissue and skeletal muscle), suggests that leptin has peripheral actions. Experimental studies have suggested that leptin can impair insulin signaling in skeletal muscle and adipocytes, changing the lipid synthesis and degradation ratio [1, 9, 10]. Thus, the dysregulation of leptin actions, e.g., changes in leptin receptor expression in adipose tissue, may influence obesity and other metabolic disorders development, such as insulin resistance and type II diabetes [1, 11]. Circulating leptin is correlated with adipose tissue mass, but may decrease in a short-term fasting, and increase after refeeding [12–14], despite a minimal change in adipose pads, suggesting that recent changes in energy balance have a major influence on leptin levels [15]. Furthermore, dietary macronutrient content may affect leptin concentration [13]. It was observed that leptin responses to macronutrient composition in a different way, suggesting an increase in leptin levels after a carbohydrate meals intake [16], with changes in leptin mRNA expression [17].

With changes in energy expenditure, exercise fuels flux, and systemic hormone concentration contributes to leptin regulation [15]. However, the effects of physical training on the regulation of leptin are conflicting. Although some authors found no change in hormone levels with exercise training, it is well established that chronic exercise results in a change in leptin levels [18, 19]. The pathways involved may be related to alterations in the long form of the leptin receptor OB-Rb [20] at the central level. Zhao et al. [21] demonstrated that endurance exercise during a 9-week period activates the same signaling pathways induced by leptin in the rat hypothalamus, suggesting a mimetic effect of training in relation to the hormone in the hypothalamic region, resulting in a reduction in serum leptin after exercise. However, there are no reports concerning the effects of exercise on adipocyte leptin receptors.

Adipose tissue lipolysis can be extracellular, a process which is mediated by lipoprotein lipase (LPL), or intracellular, a process which is mediated by catecholamine [22], and some studies have suggested that the sympathetic nervous system is the primary physiological regulator for leptin synthesis in this tissue [23]. There are studies reporting that the exercise-induced oxidation of energy substrates, such as glucose and fatty acids, changes the leptin concentration, suggesting that the reduction in hormone levels is due to changes in availability or nutrient flux [24, 25]. As mentioned above, both diet composition and exercise influence serum leptin; however, there are no data on their combined long-term effects on leptin metabolism in WAT.

Indeed, leptin functions in the WAT has been essential for modulating adipocyte metabolic functions [10], upregulating fat oxidation [26] and decreasing lipogenesis [27]. Some authors consider the leptin paracrine actions to be important for the metabolic system of the entire body [1]. The knowledge of the factors that contribute with leptin receptor expression in WAT as well as understanding the effect of diet macronutrient composition and exercise training on leptin levels may assist us to understand the impact of lifestyle on body weight regulation [15].

The aim of this study was to investigate the effects of physical training (during 4- and 8-week periods) in rats fed an HSD on the circulating levels of leptin, its peripheral actions, measuring the *Lep* and *OB*-*Rb* mRNA levels and hypertrophy effects in rWAT. Previous studies performed by our group demonstrated that rats fed an HSD and subjected to running training for an 8-week period had the relationship between the *Ucp1*/*Ucp3* mRNA levels impaired. This change may result in lower energy efficiency and may also explain the increase in the adipose index observed in these animals because exercise training blocked the HSD-induced up-regulation of UCP1 expression in iBAT and up-regulated the *Ucp3* mRNA levels in muscle tissues [28]. Thus, the hypothesis examined in this study was that exercise does not attenuate the effects of an HSD on fat cell size and lipid content, and the mechanism of action involved in this process is mediated by leptin through its receptors in adipocytes, which involves the regulation of the TAG synthesis and storage/lipolysis pathways. Because the action of leptin on WAT may regulate fatty acid oxidation, non-esterified fatty acid (NEFA) circulating levels, catecholamine and *Ucp2* mRNA expression were also analyzed in rWAT. The retroperitoneal fat pad was chosen because it is one of the major sites of leptin production in rodents [29], and it may be related to the metabolic complications of obesity [30]. Furthermore, the retroperitoneal fat pad is very responsive to diet interventions [28, 31].

## Methods and Materials

### Animals

Four-week-old weaned male Wistar rats were housed in individual cages under controlled light (05.00–19.00 h) and temperature (24.0 ± 2.0 °C) conditions with water and rat chow provided ad libitum. Before starting the training, the animals were randomly divided into the following groups: (1) sedentary rats fed with a standard chow diet (S-STD, sedentary-standard diet; *N* = 12), (2) trained rats fed with a standard chow diet (T-STD, trained-standard diet; *N* = 12), (3) sedentary rats fed with a high-sugar diet (S-HSD, sedentary-high-sugar diet; *N* = 12), and (4) trained rats fed with a high-sugar diet (T-HSD, trained-high-sugar diet; *N* = 12). The Ethics Committee of the Federal University of Minas Gerais for the Care and Use of Laboratory Animals (protocol 192/08) approved all the experimental procedures, and they were conducted in accordance with the Committee’s Guiding Principles Manual.

### Diet

The animals were fed for 4- and 8-week periods with an HSD (68 % carbohydrates, S-HSD and T-HSD groups) consisting of 33 % standard chow (Nuvilab CR1, Nuvital, Brazil), 33 % condensed milk and 7 % sucrose by weight (the remainder was water) [31]. The control groups were fed a standard chow (STD). The percent composition of each diet has been previously published [28].

Rats had calorie intake and body weight measured once a week during the experimental period. After 4- and 8-week periods, the weekly food intake was multiplied by the energy density for the STD (12.22 kJ/g) and the HSD (13.31 kJ/g), to calculate the energy intake.

### Exercise Training

After weaning, rats underwent exercise training on a motor-driven treadmill (Gaustec, Contagem, Brazil), using the same protocol published previously [28]. The animals were conditioned for exercise for 5 days (10 m/min on a 5 % incline for 5 min/day), and then, they were subjected to a workload running test [32]. This test was repeated at the end of the 4th and 8th weeks of training, to assess improvement in running performance [28, 33, 34]. Workload (*W*) was calculated as *W* = body weight (kg) × time to fatigue × treadmill speed (m/min) × sin *θ* (treadmill inclination) [21]. After the last test, all groups (4- and 8-week periods) increased their performance, regardless of the diet (Table 1).

**Table 1:** Characteristics of rats fed an STD or HSD for 4- and 8-week periods

	Body weight (g)	Calorie intake (kJ)	Adipose index	*W*	CS activity (μmol/mL/min)
4-week period					
S-STD (*N* = 6)	254.0 ± 9.2	334.7 ± 5.9	2.7 ± 0.2	32.2 ± 2.0	6.8 ± 0.9
S-HSD (*N* = 6)	273.0 ± 10.9	382.4 ± 6.8	3.9 ± 0.2*	36.8 ± 2.6	6.9 ± 0.6
T-STD (*N* = 6)	234.5 ± 10.6	289.5 ± 5.3	2.8 ± 0.7	47 ± 6.3^#^	7.9 ± 1.2
T-HSD (*N* = 6)	262.9 ± 10.2	371.5 ± 7.5	4.6 ± 0.5*	53.1 ± 3.5^#^	6.4 ± 0.8
8-week period					
S-STD (*N* = 6)	387.3 ± 11.1^a^	264.8 ± 8.4	2.8 ± 0.3	51.0 ± 5.0^a^	8.8 ± 1.9
S-HSD (*N* = 6)	404.3 ± 32.3^b^	259.8 ± 8.3	4.6 ± 0.6*	48.6 ± 4.7^b^	11.3 ± 1.7*^, b^
T-STD (*N* = 6)	363.8 ± 16.8^c^	254 ± 8.0	1.5 ± 0.2^#, c^	77.9 ± 11.0^#, c^	14.9 ± 1.3^#, c^
T-HSD (*N* = 6)	412.4 ± 6.7^d^	264.8 ± 8.4	4.3 ± 0.5*	77.5 ± 11.5^#, d^	18.4 ± 1.1*^, #, d^

The data are expressed as means ± SD. All comparisons were performed by two-way ANOVA (Bonferroni test). *P* values <0.05 were considered statistically significant

*S-STD* sedentary-standard diet, *S-HSD* sedentary-high-sugar diet, *T-STD* trained-standard diet, *T-HSD* trained-high-sugar diet, *W* workload, *CS* citrate synthase

* Statistically significant differences compared with its STD control

^#^Statistically significant differences compared with its untrained control (S-STD or S-HSD)

^a^Statistically significant differences compared with the S-STD group (4-week period)

^b^Statistically significant differences compared with the S-HSD group (4-week period)

^c^Statistically significant differences compared with the T-STD group (4-week period)

^d^Statistically significant differences compared with the T-HSD group (4-week period)

The exercise training protocol consisted of daily running sessions with gradual intensity increases (10 m/min/30 min period and was increased until the rats were able to run at 25/60 m/min), as previously described [32, 35]. The achievement of this exercise intensity and duration resulted in the enhancement in citrate-synthase activity, for the 8-week period. For the 4-week period, exercise training was interrupted halfway through the protocol, i.e., when the rats were able to run at 15 m/min (5 % incline) for 60 m/min, to determine whether a 4-week period was sufficient to detect the beginning of changes induced by endurance training for the parameters assessed. All groups were subjected to consistent handling procedures. The S-STD and S-HSD groups underwent running exercises for 2 min, following the same physical training schedule. Running procedures were performed between 08.00 and 11.00 h, at 23 ± 1 °C [36, 37].

### Euthanasia

The animals were decapitated 24 h after completion of the physical training protocol, and the sera, adipose tissues (retroperitoneal, epididymal, and inguinal) and soleus muscles were collected. Rats were not under fasting conditions. Retroperitoneal fat pads were immediately removed, weighed, snap-frozen in liquid nitrogen and stored at −70 °C until further analysis. To evaluate to the development of obesity, the adiposity index was calculated as 100 × (sum of fat pad weights)/(body weight) [38]. To calculate the fat pad weight, all WAT removed were used.

### Adipocyte Size and Lipid Content

Retroperitoneal fat pad sections were fixed in 3.7 % formaldehyde, embedded in paraffin, sliced to 5-μm thickness with a microtome, and stained with hematoxylin-eosin for each trial group. Sections were microscopically visualized with a 40× objective and images were digitized with a Leica DFC340FX microcamera connected to a Leica DM5000B microscope; all images were analyzed using the image processing and analysis software Leica Qwin V3 at Multiuser Laboratory of Núcleo de Pesquisas em Ciências Biológicas of UFOP. The number and the area of adipocytes were assessed by quantification in the retroperitoneal fat covering a total area equal to 7.5 × 10^5^ μm^2^. The adipocytes were examined blind by the experimental group.

The lipid content was measured according to the Folch method [39], and the data were expressed as grams of lipids per relative rWAT weight (i.e., rWAT weight divided by body weight).

### Total RNA Preparation and Lep, OB-Rb, and Ucp2 Expression Analysis by qRT-PCR

Total RNA was obtained from rWAT using Trizol™ reagent (Invitrogen, São Paulo, Brazil) and chloroform (Sigma-Aldrich) for extraction, and it was purified using the SV total RNA Isolation System kit (Promega™), according to the manufacturer’s protocol. Total RNA was quantified and the integrity was verified by electrophoresis on a 1.2 % agarose formamide-TBE gel. The total RNA was treated with RNase-free DNase I, for 30 min and measured at 260 nm. Ratios above 1.8 were considered suitable for the quantification of gene expression [40].

Total RNA (one microgram) was reverse-transcribed using random primers from the High Capacity RT-PCR System (Applied Biosystems) according to the manufacturer’s protocol. Complementary DNAs (cDNAs) encoding for *Lep*, *OB*-*Rb*, *Ucp2* and *rRNA 18S*, which was used as an endogenous control, were obtained by PCR amplification using SYBR^®^ Green PCR Master Mix (Applied Biosystems). Primers were designed using Gene Runner™ Software, and the following sequences were deposited in the Rat Genome Database (RGD): *Lep* [RGB: NM_013076.2] (forward: 5′ CAGGCTCTCTGGCTTCTG 3′ and reverse: 5′ GAGACCTCCTCCATCTGCTG 3′), *OB*-*Rb* [RGB: NM_012596.1] (forward: 5′ GAACCTGTGAGGATGAGTG 3′ and reverse: 5′ CACTGGCTGACAGAACTATG 3′), *Ucp2* [NM_019354.2] (forward: 5′ CTGGCGGTGGTCGGAGATAC 3′ and reverse: 5′ GGGCAACATTGGGAGAGGTC 3′) and *18S* *rRNA* [RGB: X01117.1] (forward: 5′ GTAAGTGCGGGTCATAAG 3′ and reverse: 5′ CCATCCAATCGGTAGTAGC 3′). The reactions were performed under the standard conditions from Applied Biosystems ABI 7300 Real-Time PCR system. The primers had their efficiency evaluated from serial dilutions, and the specificity of the products obtained was confirmed by analysis of dissociation curves from the amplified products. Target gene expression was normalized against the *rRNA 18S* transcript according to the 2^−Δ*C*q^ method [41]. For the investigated transcripts, three biological replicates were performed, and the relative expression (2^−ΔΔ*C*q^ method) was measured using the S-STD group as the calibrator sample, where its expression was considered as the 1× control index for comparison with target genes.

### Serum Leptin, Non-Esterified Fatty Acid (NEFA) and rWAT Total Catecholamine

Serum leptin concentrations were measured by radioimmunoassay (*Rat Leptin RIA*, Linco Research, RL-83K) according to the manufacturer’s protocol. Serum NEFA were determined by the colorimetric method using a *NEFA* (Randox ⊂ ∈, FA115) kit according to the manufacturer’s protocol. The rWAT total catecholamines were measured by the tri-hydroxyindole fluorimetric method, using epinephrine (Adren™, Hipolabor) diluted in 10 % acetic acid as a standard, as previously described [42].

### Enzymatic Assay

The biomarker of oxidative metabolism in soleus muscle tissues was measured using a Citrate Synthase Assay kit (Sigma-Aldrich). Briefly, tissue samples were homogenized (50 mM Tris–HCl, 1 mM EDTA, and 0.01 mM phenylmethylsulfonyl fluoride; pH 7.4) using a Polytron homogenizer, and then the homogenates were centrifuged (725×*g* for 10 min at 4 °C). The supernatant was decanted, and the citrate synthase activity was assayed according to the manufacturer’s protocol.

### Statistical Analysis

Statistical analyses were performed using the Graph Pad Prism (version 5.0) software (Irvine, CA, USA). The sample size was determined, considering the minimum difference between the mean and standard deviation of error with a power of 0.9 and a significance level (*α*) of 0.05. The highest estimated size to assess our outcomes was chosen (*N* = 6). The Shapiro–Wilk test was used to verify data normalization. The data are reported as the means ± standard deviations (SD). Differences between groups were evaluated using two-way ANOVA followed by the Bonferroni test. *P* values <0.05 were considered statistically significant.

## Results

### Characteristics of Sedentary and Trained Animals

Caloric intake and body weight did not differ among groups, even those fed an HSD (Table 1). However, the adipose index was influenced by diet (*P* < 0.001) for both periods. The HSD increased the adipose index over a 4-week period, while its association with exercise did not prevent this increase in the same exercise period (compared with T-STD). We also observed that exercise induced a 45 % decrease in the adipose index over the 8-week period. With regard to changes in citrate-synthase activity in the soleus muscle, our results demonstrated an increase in the trained animals in the 8-week period group regardless of diet (Table 1).

### The Effects of Diet and Endurance Training on rWAT: Fat Cell Size and Number, Lipid and Catecholamine Content

**Fig. 1:**
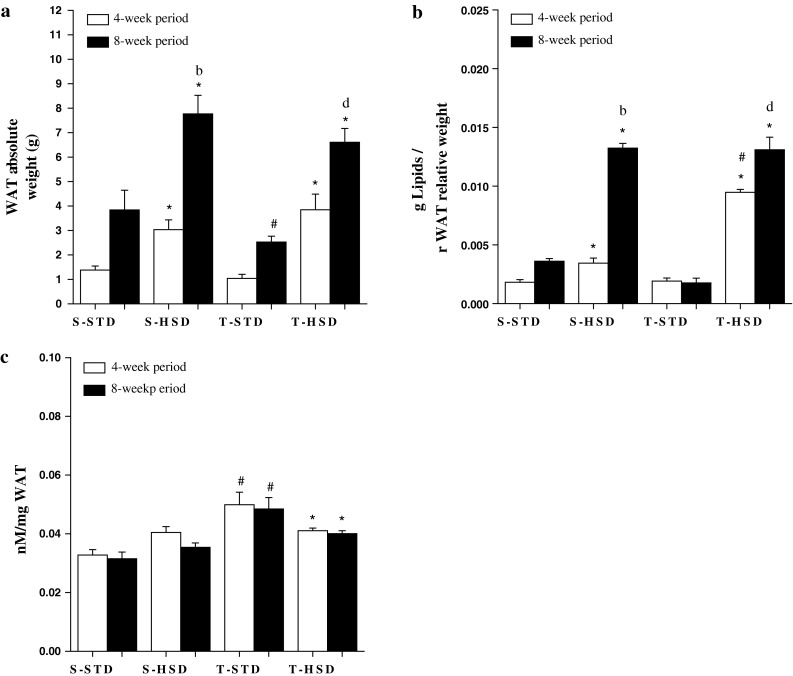
The effects of a high-sugar diet and exercise training on rWAT over 4- (*white bars*) and 8-week (*black bars*) periods. **a** Absolute rWAT weights. **b** The lipid content was measured according to the Folch method. **c** The total catecholamine content (nM) per mg of rWAT was measured using the tri-hydroxyindole fluorimetric method. The data are expressed as the means ± SD. The effects of diet and/or exercise were compared using two-way ANOVA (Bonferroni test), and *P* values <0.05 were considered statistically significant. *Statistically significant differences compared with its STD control, ^#^statistically significant differences compared with its untrained control (S-STD or S-HSD). ^a^Statistically significant differences compared with the S-STD group (4-week period), ^b^statistically significant differences compared with the S-HSD group (4-week period), ^c^statistically significant differences compared with the T-STD group (4-week period) and ^d^statistically significant differences compared with the T-HSD group (4-week period). *S-STD* sedentary-standard diet, *S-HSD* sedentary-high-sugar diet, *T-STD* trained-standard diet, *T-HSD* trained-high-sugar diet

As shown in Fig. 1a, the HSD influenced the rWAT weight from the 4-week period, increasing the rWAT mass by ~115 % in the S-HSD group (compared with the S-STD group), while its association with exercise did not prevent this alteration in the T-HSD group. However, endurance training reduced the fat pad in the T-STD group in the 8-week period.

**Fig. 2:**
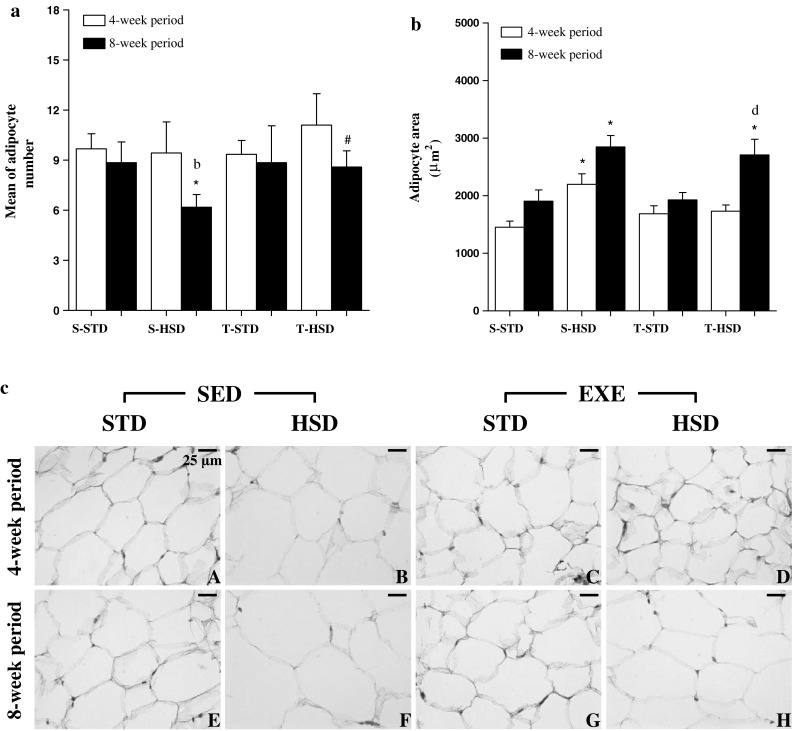
The effects of a high-sugar diet and exercise training on adipocyte number and size over 4- (*white bars*) and 8-week (*black bars*) periods. **a** The adipocyte number and **b** size among the groups were microscopically visualized in a fixed area (7.5 × 10^5^ μm^2^) and photographed with a digital camera. The data are expressed as the means ± SD. The effects of diet and/or exercise were compared using two-way ANOVA (Bonferroni test), and *P* values <0.05 were considered statistically significant. **c** Histological sections of adipocytes’ average area, hematoxylin–eosin. *Bar* 25 μm. *Statistically significant differences compared with its STD control, ^#^statistically significant differences compared with its untrained control (S-STD or S-HSD). ^a^Statistically significant differences compared with the S-STD group (4-week period), ^b^statistically significant differences compared with the S-HSD group (4-week period), ^c^statistically significant differences compared with the T-STD group (4-week period) and ^d^statistically significant differences compared with the T-HSD group (4-week period). *S-STD* sedentary-standard diet, *S-HSD* sedentary-high-sugar diet, *T-STD* trained-standard diet, *T-HSD* trained-high-sugar diet

The HSD also induced adipose tissue hypertrophy, reflected in a lower number of adipocytes per selected area (Fig. 2a), rather than changes in cell number, during the 8-week period (Fig. 2a). Its association with exercise had an inverse effect: there was an increase of ~35 % in the number of adipocytes per selected area in the T-HSD group (compared with the T-STD).

The adipocyte size, which increased ~50 % during the 4-week period, was influenced by the HSD, and endurance training was unable to prevent this increase during the 8-week period (Fig. 2b). The lipid content was similarly increased by the HSD from the 4-week period, and this increase was not prevented by exercise. The difference observed in lipid content between the S-HSD and T-HSD groups in the 4-week period disappeared in the 8-week period (Fig. 1b).

As shown in Fig. 1c, the total catecholamine in the rWAT was influenced by exercise (*P* < 0.001), which increased the catecholamine content in rWAT in the 4-week period. However, in trained rats fed an HSD, the catecholamine content was lower. The same pattern was observed for the 8-week period.

### Effects of Diet and Endurance Training on Leptin Levels: Lep and Ob-Rb mRNA Expression

**Fig. 3:**
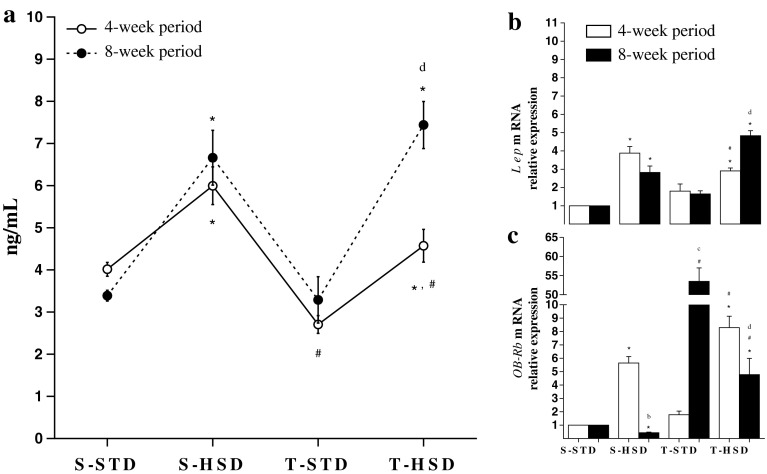
The effects of a high-sugar diet and exercise training on leptin over 4- (*white*) and 8-week (*black*) periods. **a** Leptin serum levels (ng/mL). The data are expressed as the means ± SD. **b** The Lep and **c** OB-Rb mRNA levels in rWAT. The gene expression profiles of the S-STD, S-HSD, T-STD and T-HSD groups were evaluated using the 2^−ΔΔ*C*q^ method. rRNA 18S was used as a reference gene, and the S-STD group was used as a calibration sample, where its expression was considered as the 1× control index for comparison with the other groups. The effects of diet and/or exercise were compared using two-way ANOVA (Bonferroni test), and *P* values <0.05 were considered statistically significant. *Statistically significant differences compared with its STD control, ^#^statistically significant differences compared with its untrained control (S-STD or S-HSD). ^a^Statistically significant differences compared with the S-STD group (4-week period), ^b^statistically significant differences compared with the S-HSD group (4-week period), ^c^statistically significant differences compared with the T-STD group (4-week period) and ^d^statistically significant differences compared with the T-HSD group (4-week period). *S-STD* sedentary-standard diet, *S-HSD* sedentary-high-sugar diet, *T-STD* trained-standard diet, *T-HSD* trained-high-sugar diet

As shown in Fig. 3a, the HSD influenced the leptin levels, increasing by 65 % in the S-HSD group from the 4-week period (compared with the S-STD group). Although the HSD association with endurance training increased the leptin levels (i.e., T-HSD compared with the T-STD group), the exercise during the 4-week period decreased the leptin levels when compared with the sedentary controls. However, the effect of endurance training in rats fed an HSD on leptin levels disappeared in the 8-week period.

As shown in Fig. 3b, the HSD increased the *Lep* mRNA expression fourfold during the 4-week period, and exercise did not block this increase. Interestingly, exercise did not alter the *Lep* mRNA expression in either period.

The relative expression of *OB*-*Rb* mRNA in rWAT was directly affected by the HSD (Fig. 3c). Similar to *Lep* gene expression, the HSD increased the *OB*-*Rb* mRNA expression sixfold during the 4-week period. Conversely, in rats fed an HSD, exercise increased the *OB*-*Rb* mRNA levels (i.e., T-HSD compared with the T-STD and S-HSD groups) during the same period. However, in the 8-week period, the HSD decreased the *OB*-*Rb* mRNA expression twofold, and exercise upregulated the transcript levels (T-STD group). In rats fed an HSD, exercise downregulated the relative expression of *OB*-*Rb* mRNA (i.e., T-HSD compared with the T-STD group and the 4-week period); however, this association increased the transcript levels eightfold (T-HSD group) compared with the sedentary control (S-HSD group).

### The Effects of Diet and Endurance Training on NEFA and Ucp2 mRNA Levels

**Fig. 4:**
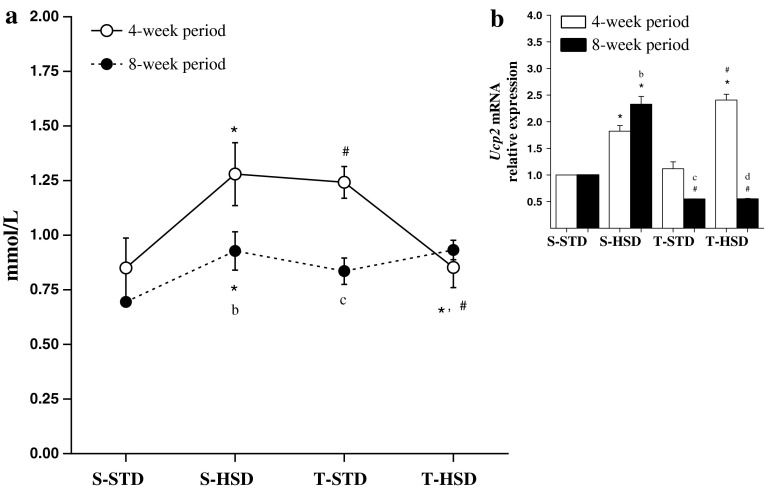
The effects of a high-sugar diet and exercise training on NEFA and Ucp2 mRNA expression in rWAT during 4- (*white*) and 8-week (*black*) periods. **a** Non-esterified fatty acid (NEFA) serum levels (mmol/L). The data are expressed as the means ± SD. **b** Ucp2 mRNA levels. The gene expression profiles were evaluated for the S-STD, S-HSD, T-STD and T-HSD groups by the 2^−ΔΔ*C*q^ method. rRNA 18S was used as a reference gene, and the S-STD group was used as a calibration sample, where its expression was considered as the 1× control index for comparison with the other groups. The effects of diet and/or exercise were compared using two-way ANOVA (Bonferroni test), and *P* values <0.05 were considered statistically significant. *Statistically significant differences compared with its STD control, ^#^statistically significant differences compared with its untrained control (S-STD or S-HSD). ^a^Statistically significant differences compared with the S-STD group (4-week period), ^b^statistically significant differences compared with the S-HSD group (4-week period), ^c^statistically significant differences compared with the T-STD group (4-week period) and ^d^statistically significant differences compared with the T-HSD group (4-week period). *S-STD* sedentary-standard diet, *S-HSD* sedentary-high-sugar diet, *T-STD* trained-standard diet, *T-HSD* trained-high-sugar diet

As shown in Fig. 4a, the HSD influenced the NEFA levels, which increased by 50 % in the S-HSD group during the 4-week period (S-HSD compared with the S-STD group); however, this effect was reduced in the 8-week period. Exercise increased the NEFA levels by 45 % during the 4-week period, and its association with the HSD decreased these levels during the same period (T-HSD compared with the T-STD and S-HSD groups). The effects of the HSD (S-HSD group) and exercise (T-STD group) were reduced in 8-week compared with 4-week rats.

As shown in Fig. 4b, the HSD increased *Ucp2* mRNA expression twofold in the 4-week period, and exercise did not prevent this increase (T-HSD compared with the T-STD and S-HSD groups). Exercise decreased the transcript levels by ~2 fold in the 8-week period, while its association with the HSD also downregulated *Ucp2* mRNA expression during the same period (T-HSD compared with the S-HSD group).

## Discussion

The main finding of this study was that rats undergoing endurance training and fed with a STD, upregulate *OB*-*Rb* mRNA in rWAT, suggesting a leptin autocrine mechanism that promotes tissue lipolysis and assists catecholamines in reducing the fat pad during an 8-week period. However, when the rats were fed with an HSD, the increase in the *OB*-*Rb* mRNA expression and the catecholamine levels observed in the T-STD group were reduced, resulting in a high level of adiposity and hyperleptinemia. In addition, the combined effect of training and an HSD on fatty acid metabolism was age dependent, decreased the circulating NEFA and upregulated the *Ucp2* mRNA expression in the 4-week period, while it downregulated the *Ucp2* mRNA expression in the 8-week period without changes in NEFA levels.

Our results demonstrated that the effects of an HSD on leptin mRNA expression were reflected in the protein levels over a 4-week period. The metabolic properties of adipose tissue, such as the secretion of leptin, are related to the amount of fat stored in specific depots; in other words, the increase in leptin levels is proportional to the increase in fat mass [43]. This increase may be a consequence of a diet-induced lipogenic stimulus, which is represented by an increase in the adipocyte size, lipid content, and rWAT observed in these animals. Is noteworthy that there was fewer cells within the selected area due to increase in cells volume. Koutsari et al. [15] demonstrated that, in women, leptin concentrations are more responsive after HSD, suggesting a critical role for insulin in the regulation of the leptin production in response to the type of carbohydrates provided by this type of diet [15]. However, the literature reports a hyperleptinemia phenotype in animal models consuming high-fat diets without exploring the effects of an HSD on metabolic complications arising from adipose tissue for short and long durations.

Taking into account leptin as an energy balance regulator [44], an increase in its level induced by an HSD may be explained by the occurrence of a positive energy balance, which results in increasing the adipocyte lipid content, thereby increasing its size. The increase in fat cell size causes an expansion of rWAT and the upregulation of *Lep* mRNA, resulting in increased circulating levels. In this sense, the hormone does not cause changes in food intake and fat accumulation because the constant lipogenic stimulus (HSD) could result in central resistance to leptin [45]. However, this phenomenon must be investigated further.

Considering the central-resistance mechanism induced by leptin, questions arise regarding the adipokine pleiotropic effect including why the high circulating hormone concentration does not stimulate rWAT lipolysis because the universal distribution of their receptors may allow for an independent effect of the sympathetic nervous system [46], resulting in body fat reduction.

It is noteworthy that our data suggest an age-dependent effect of HSD on *OB*-*Rb* mRNA expression. In young animals (4-week period), there was an upregulation of *OB*-*Rb* mRNA in rWAT, suggesting an increased susceptibility to leptin as an adaptive measure to prevent the adipose tissue increase induced by an HSD in the short term. This hypothesis is rather attractive because the comparison of the lipid content between the two phases demonstrated a significant reduction during the 4-week period; however, the NEFA levels were increased in the S-HSD group, and leptin-mediated lipolysis does not result in elevated circulating NEFA [9]. Moreover, it may be necessary to prove whether the increase in the *OB*-*Rb* mRNA levels is reflected by its protein expression in order to elucidate the actual mechanism.

Nevertheless, the downregulation of *OB*-*Rb* mRNA expression in rWAT was observed in the 8-week period, suggesting the occurrence of a blockage in the leptin-induced paracrine action in adipocytes induced by an HSD, leading to the accumulation of triacylglycerol (TAG). Previous studies reported the occurrence of a leptinergic block in adipose tissue after high-fat diet administration concomitant with suppressor of cytokine signaling-3 (SOCS-3) upregulation and a gradual reduction in *OB*-*Rb* mRNA with hyperleptinemia increases, and the receptor levels completely disappeared after 19 weeks of this diet. The authors suggested that the combination of leptin blockage at the level of the leptin post-receptor and receptor minimize the leptinergic potential interference with fat stores. The *OB*-*Rb* mRNA downregulation may be mediated by insulin, because it is a coupled phenomenon with an increased adipocyte lipid content and upregulation of leptin expression [10]. Taken together, these results suggest the existence of a system that is mediated by OB-Rb that regulates energy balance in adipocytes that favors the development of diet-induced obesity [1, 10]. This is the first study to highlight the effects of an HSD on *OB*-*Rb* mRNA expression in rWAT, and to report that diet macronutrient composition may determine the propensity of individuals to obesity. However, future experiments are needed to elucidate the exact mechanism by which this process occurs.

With regard to endurance training, in this study, we observed a decrease in leptin plasma levels without changes in *Lep* and *OB*-*Rb* mRNA expression for a 4-week period. Although opinions about the effects of exercise on leptin levels are conflicting, some suggest that the serum hormone levels may be sensitive to rapid changes in training load, suggesting that the exercise duration (< or ≥60 min) and training period (< or ≥12 weeks) influence these levels [24, 47]. Moreover, previous studies demonstrated that lower leptin levels are observed after long-term exercise (≥60 min), which stimulates the release of NEFA, or after exercise that generates energy expenditure greater than 800 kcal [47]. Considering that our animals trained for 60 min (long-term exercise) and the NEFA levels were increased in the T-STD group (4-week period), our results are in agreement with this work, suggesting that the decrease in leptin levels was a result of this increase in circulating NEFA promoted by the exercise in this period.

In the 8-week period, upregulation of *OB*-*Rb* mRNA expression and decreases in NEFA levels and rWAT weight were observed. It is noteworthy that the adipose tissue lipolysis could be mediated by catecholamine, via β3-adrenoceptors, or leptin, via OB-Rb receptors. As mentioned above, the leptin-induced lipolysis does not increase NEFA levels [9]. Our results suggest that endurance training stimulates the leptin autocrine action, via upregulation of its receptor, and favors tissue lipolysis, decreasing the adiposity index observed in these animals after the 8-week period. Despite that fact that our data were related only to mRNA levels, this is the first report that proposes a leptin-peripheral mechanism that controls body fat, suggesting the beneficial effects of exercise on lipolysis/lipogenesis in rWAT. Additionally, our results do not exclude the possibility of a centrally mediated effect of exercise, because Kimura et al. [20] demonstrated spontaneous exercise-regulated leptin expression in mice after a 12-week period, reducing body fat, and the leptin concentration induced downregulation of *OB*-*Rb* mRNA in the hypothalamus [20]. However, it is noteworthy that exercise bouts with greater energy expenditure influence the circulating hormone levels, and the training period might be related to this factor [47].

Even during the 8-week period, the downregulation in *Ucp2* mRNA suggests an increase in energy efficiency in trained rats fed an STD, corroborating a previous study from our group in which we demonstrated that training enhances the relationship between the *Ucp1/Ucp3* mRNA levels [28].

In trained rats fed an HSD, the effect on adipose tissue metabolism was also age dependent. For animals belonging to the 4-week period group, although the *Lep* mRNA expression and serum protein levels were greater than those observed in the T-STD group, exercise restricted the increase induced by the HSD during the same period. Koutsari et al. [15] observed that moderate daily exercise suppresses circulating leptin concentrations in fasting and postprandial individuals without changing the body composition of women who consumed a short-term high-carbohydrate diet. These authors suggested that insulin plays a crucial role in this mechanism because it stimulates the secretion of leptin due to the increase in the uptake of glucose by adipocytes and metabolism. Although the serum leptin in our animals was not measured during fasting, our results reinforce the idea that there was a relocation of the rWAT glucose metabolism in the T-HSD group (4-week period) because the literature reports that skeletal muscle plays a major role in ingested carbohydrate utilization (during the postprandial period), whereas fat tissue plays a secondary role [48]. Thus, after exercise in rats fed an HSD, our results suggest that a higher proportion of ingested carbohydrates was absorbed by skeletal muscle, reducing the exposure of rWAT to glucose, which reduced leptin secretion by adipocytes in the short term [15]. However, further experiments are required to confirm this hypothesis.

Additionally, the lower rWAT lipid and catecholamine content (compared with the 4-week period and exercise, respectively), the upregulation of the *OB*-*Rb* and *Ucp2* mRNAs, and the lower amount of circulating NEFA in the T-HSD group suggest that in exercised young rats fed an HSD for a 4-week period, there is leptin-mediated lipolysis in adipose tissue. Although our findings only refer to mRNA levels, this hypothesis is rather attractive because hyperleptinemia causes a reduction in fat mass without causing an elevation in NEFA, suggesting that they are oxidized inside adipocytes [9]. Reinforcing our hypothesis, the serum NEFA and *Ucp2* mRNA regulation in the T-HSD group were lower than those observed in the T-STD group, suggesting the occurrence of leptin-mediated NEFA oxidation in adipocytes. However, over a long term (8-week period), the effect of leptin on rWAT metabolism was adverse. The downregulation of *OB*-*Rb* mRNA suggests that the lipolysis rate in rWAT decreased, contributing to tissue expansion, the upregulation of *Lep* mRNA and elevated serum leptin. Moreover, the rWAT total catecholamine levels were reduced during the same period. Jocken et al. [22] demonstrated that the effect of catecholamine-induced lipolysis impairment contributes to the development or maintenance of increased fat stores and obesity, which may have contributed to the fat pad expansion and increases in leptin expression and secretion during the 8-week period. Our results suggest that in trained rats fed an HSD for an 8-week period, there is an inhibition of fatty acid oxidation by dietary glucose, which is corroborated by the downregulation of *Ucp2* mRNA in the T-HSD group. Data from our group demonstrated that in trained rats fed an HSD, a decrease occurs in the relationship between *Ucp1/Ucp3* mRNA, contributing to a worsening of energy efficiency and culminating with an increase in the adiposity index observed during the same period [28].

In conclusion, the results of this study suggest that an HSD induces an increase in leptin expression in rWAT, with the downregulation of *OB*-*Rb* mRNA after an 8-week period and favors TAG synthesis and storage, which culminates with elevated serum leptin. Regarding exercise, there was a reduction in the leptin serum with no change in the *Lep* mRNA expression and an upregulation in *OB*-*Rb* mRNA, suggesting a reduction in adipocytes via leptin-mediated lipolysis after an 8-week period. In exercised rats fed an HSD, despite the increased *OB*-*Rb* mRNA levels (relative to a sedentary control), TAG synthesis and storage overlaps with lipolysis, favoring the development of fat stores and the upregulation of *Lep* mRNA and plasma protein in adult rats (8-week period).

## Abbreviations

HSD: High-sugar diet
RGD: Rat genome database
OB-Rb: Leptin receptor
rWAT: Retroperitoneal white adipose tissue
S-HSD: Sedentary-high-sugar diet
S-STD: Sedentary-standard diet
qRT-PCR: Quantitative reverse transcription polymerase chain reaction
T-HSD: Trained-high-sugar diet
T-STD: Trained-standard diet
Ucp2: Uncoupling protein 2
